# Direct laser writing of a new type of waveguides in silver containing glasses

**DOI:** 10.1038/s41598-017-11550-0

**Published:** 2017-09-11

**Authors:** Alain Abou Khalil, Jean-Philippe Bérubé, Sylvain Danto, Jean-Charles Desmoulin, Thierry Cardinal, Yannick Petit, Réal Vallée, Lionel Canioni

**Affiliations:** 1 University of Bordeaux, CNRS, CEA, CELIA, UMR 5107, 351 cours de la libération, F-33405 Talence cedex, France; 2 Centre d’optique, photonique et laser (COPL), Université Laval, Québec, Canada G1V 0A6; 3 University of Bordeaux, CNRS, ICMCB, UPR 0948, 87 avenue du Dr Schweitzer, F-33608 Pessac cedex, France

## Abstract

Direct laser writing in glasses is a growing field of research in photonics since it provides a robust and efficient way to directly address 3D material structuring. Generally, direct laser writing in glasses induces physical modifications such as refractive index changes that have been classified under three different types (*Type I*, *II & III*). In a silver-containing zinc phosphate glass, direct laser writing additionally proceeds via the formation of silver clusters at the periphery of the interaction voxel. In this paper, we introduce a novel type of refractive index modification based on the creation of the photo-induced silver clusters allowing the inscription of a new type of optical waveguides. Various waveguides as well as a 50–50 beam splitter were written inside bulk glasses and characterized. The waveguiding properties observed in the bulk of such silver-containing glass samples were further transposed to ribbon shaped fibers made of the same material. Our results pave the way for the fabrication of 3D integrated circuits and fiber sensors with original fluorescent, nonlinear optical and plasmonic properties. The universality of these new findings should further extend in any silver-containing glasses that show similar laser-induced behavior in terms of silver cluster production.

## Introduction

In the past decades, laser-matter interaction has occupied the scientific community due to its wide range of applications. More specifically, direct laser writing (DLW) in transparent glasses has attracted the interest of many research groups. This technique is simple and allows to directly address 3D micro-structuring inside bulk transparent materials. DLW exhibits obvious advantages over lithography techniques which is limited to 2D structuring and involve multiple steps, making DLW highly compatible for future technological transfer to advanced industrial manufacturing.

DLW consists of focusing femtosecond laser pulses inside a transparent glass substrate resulting in a permanent change in the refractive index. This new glass processing scheme based on nonlinear absorption was originally reported by *Davis et al*. in 1996^1^. The refractive index changes induced by laser-glass interaction are classified as three distinct types following a progressive energy scale^2^: *type I*, *type II & type III*. *Type I* modifications are smooth isotropic changes of the refractive index allowing the formation of waveguides^3–9^. *Type II* modifications result from a birefringent change in the refractive index associated with nano-structuration of the glass and self-organized nano-gratings formation due to the local production of moderate plasma^10–12^. Finally, *type III* modifications result in voids or disorganized damage features in the glass matrix, due to an excessive plasma production and subsequent Coulomb explosion, which are generally detrimental for the fabrication of photonic devices. In additional to glass processing, laser-written waveguides were also inscribed in crystalline material by taking advantage of the stress induced between several linear *type III* modifications^13^.

For the direct inscription of photonic components such as waveguides, one is usually interested in *type I* modifications due to the smooth nature of the index change associated with this type. In general, *type I* refractive index change is caused by the formation of color centers^14–16^, and/or the change of the glass density, which is due to the local heating and subsequent glass restructuration during the cooling stage following laser passage^17, 18^.

Recently, our group has developed a silver containing zinc phosphate glass in which DLW induces the creation of fluorescent silver clusters distributed around the laser-glass interaction voxel^19, 20^. In this paper, we present a novel type of positive refractive index modification (∆n) following irradiation with femtosecond laser pulses which highly differs from the standard *type I* modification and allows the inscription of a “new type” of waveguides. First, we characterized the fluorescent photo-induced structures using a confocal microscope, then we investigated the dependence of ∆n as a function of the irradiation conditions. Following this, 7 mm long waveguides were written inside the glass where the waveguiding aspects such as morphology, ∆n profile, near field mode profile, simulated mode profile and propagation losses were investigated. For the sake of brevity, only one single mode waveguide is presented in detail in this paper. Indeed, all modification traces exhibit rather similar waveguiding aspects. However, it should be noted that increasing ∆n results in multimode waveguides which will not be addressed in this paper. Next, a 50–50 beam splitter was successfully fabricated to illustrate the potential of this new writing process. Finally, the waveguiding properties were transposed from bulk samples to ribbon-shaped fibers both made of the same photosensitive silver-containing zinc phosphate glasses.

## Results and Discussion

**Figure 1:**
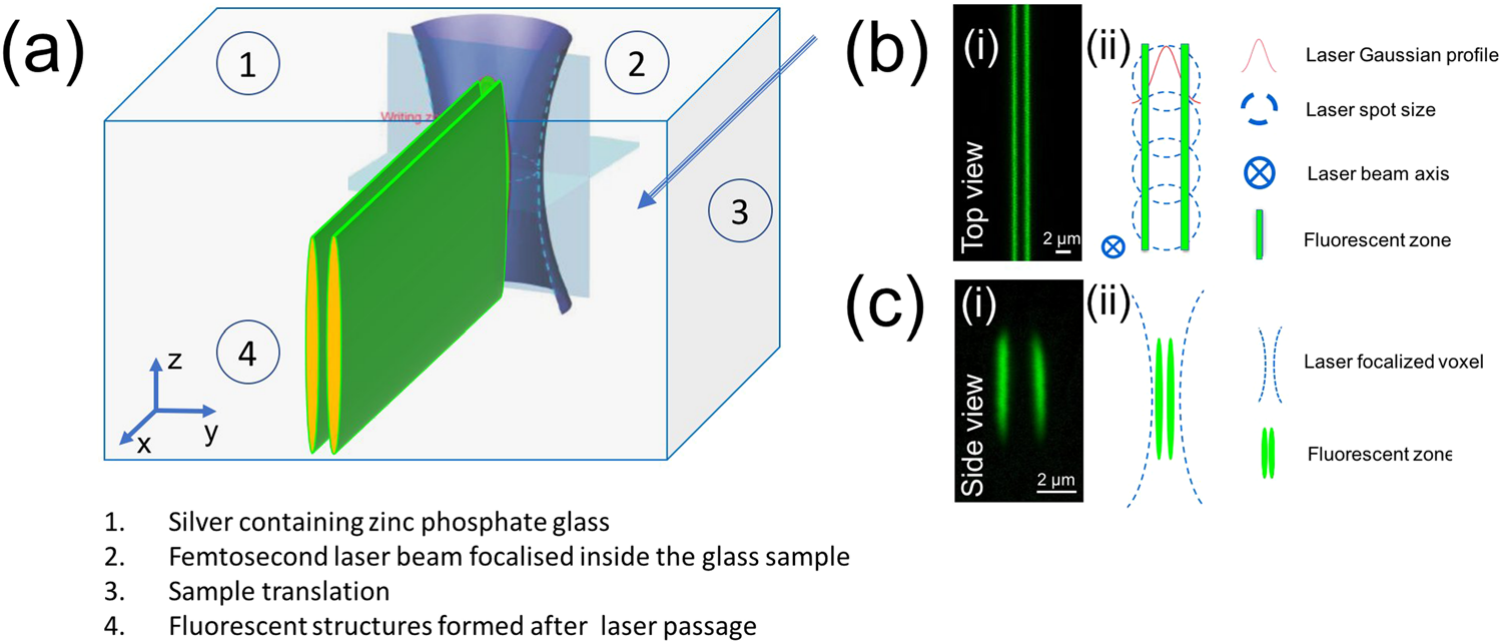
(**a**) Schematic presentation of the morphology of the structures following translation perpendicular to laser beam propagation inside silver-containing zinc phosphate glasses. Fluorescence confocal images of the (**b**.i) top view, and (**c**.i) side view. Schematic presentation of the writing process with (**b**.ii) top view and (**b**.ii) side view.

Photo-induced structures were written inside a silver-containing zinc phosphate bulk glass (4% molar of Ag_2_O) at a depth of 160 µm below the surface. The energy deposited via the nonlinear four-photon absorption processes inside the interaction voxel^20^ leads to the formation of photoinduced silver clusters Ag_m_^x+^. This is based on a pulse-to-pulse process by heat accumulation, which results in the aggregation of silver atomic and ionic species. The process of ion migration has been proposed to occur radially from the center of the beam to the edge. Therefore, a combination of electrons production, silver reduction, and silver migration leads to the formation of fluorescent silver clusters Ag_m_^x+^ at the periphery of the interaction voxel^19–22^. These clusters are characterized by two main absorption bands, typically centered at 290 nm and 345 nm and extending in the visible, and by a broad fluorescence emission covering the whole visible range^19, 20^. The glass sample was translated perpendicularly to the laser beam to form linear fluorescent structures (Fig. 1.a).

To characterize the morphology of the structures, fluorescence confocal images of the side and the top views were performed (λ_exc_ = 480 nm, λ_em_ = 550 nm). Results are presented in Figs. 1.b and 1.c. From the top view, two parallel luminescent structures are observed (Fig. 1.b.i) while the cross section (side view) shows structures distributed in two parallel planes (Fig. 1.c.i). These fluorescent double lines are typical for Ag_m_^x+^ features being photo-induced by a Gaussian profile laser beam^19, 21^ (Fig. 1.b.ii). In fact, the inscription process could be illustrated as a cumulative (i.e. many-pulses) writing process with a laser quill leaving fluorescent traces only on the edges. The absence of fluorescence in the center of the structures, i.e. the globally null budget in terms of their formation of the silver clusters in the center of the structures, is due to the high intensity of the Gaussian laser beam which results in the pulse-to-pulse photo-dissociation of the photo-induced silver clusters from the preceding laser pulse (Fig. 1.b.ii)^19–22^. The remaining clusters situated on the edges are not exposed to sufficient high laser irradiance which prevents their photo-dissociation while it allows their cumulative pulse-to-pulse growth^19, 22^ (Fig. 1.b). Furthermore, the side view (zy plane, as shown in Fig. 1.c) of the fluorescent structures reveals that the depth in the z plane is typically proportional to the nonlinear Rayleigh range associated to the multi-photon absorption^19^, as shown in Fig. 1.c.ii. This indicates that the depth of the structures can be controlled based on the microscope objective used for DLW, i.e. controlling the dimensions of the written waveguides. Such depth can also be ideally controlled by preparing the laser pulse profile to pre-compensate its phase profile in a way to further minimize the resulting spherical aberrations at the voxel, due to the refractive index mismatch between the zinc phosphate glass used here and the glass slides for which microscope objectives are designed.

**Figure 2:**
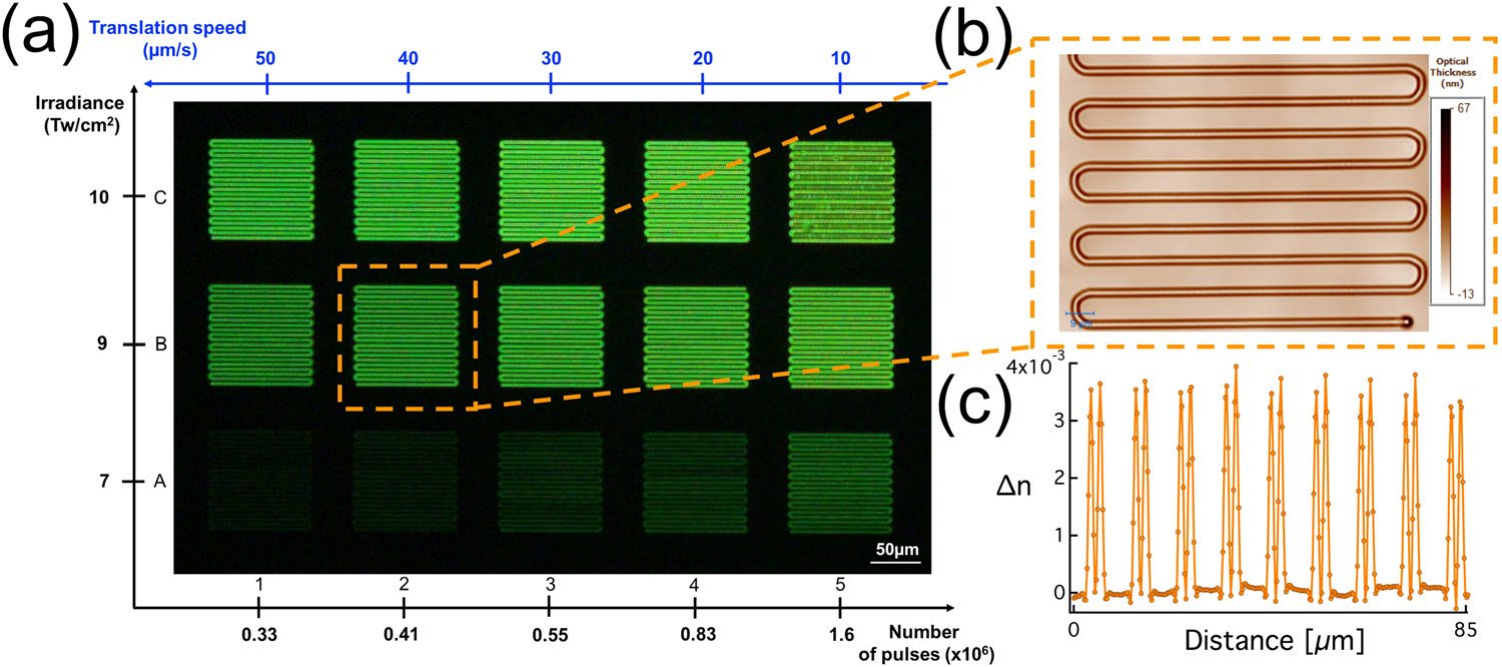
(**a**) Fluorescence image (λ_exc_ = 480 nm, λ_em_ = 550 nm) of an interaction matrix performed by DLW. Writing irradiances are presented along the vertical axis while the number of pulses appear along the horizontal axis (**b**) Blow up of the phase contrast image of the B2 structure (**c**) positive refractive index change (∆n) for the B2 structure extracted from the phase contrast image.

**Figure 3:**
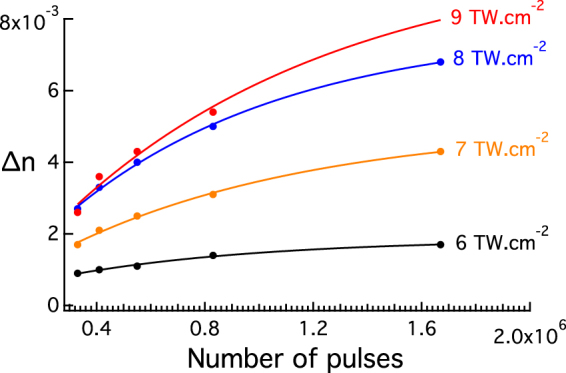
The refractive index change (∆n) as a function of the number of pulses and of laser irradiance. ∆n increases as a function of the laser parameters.

The increase of the refractive index had been briefly reported as corresponding to the creation of silver clusters in silver containing zinc phosphate glasses^23^. To further investigate this, interaction matrices of ∆n as a function of the laser parameters were realized. Structures composed of an array of 100 µm long double lines were written 160 μm below the surface with an interline spacing of 5 µm as shown in Fig. 2.a. The number of pulses deposited as well as the laser irradiance were changed during the laser writing, from 0.3 × 10^6^ to 1.7 × 10^6^ deposited pulses and from 6 to 10 TW/cm^2^, respectively, as shown in Figs 2 and 3. Top view phase contrast images of the written structures reveal a positive phase shifting spatially correlated with the distribution of the silver clusters as shown in Fig. 2.b for the structure labelled B2. Based on the phase contrast images and knowing the structures’ depth, the profile of ∆n can be retrieved, which is correlated to the spatial distribution of the silver clusters showing two positive peaks for every laser passage (Fig. 2.c). This is supporting the idea that the photo-induced silver clusters are responsible for the ∆n in this case.

Our study shows that the ∆n increases as a function of both the number of deposited pulses as well as the laser irradiance (Fig. 3). This result is expected, since a higher number of deposited pulses contributes to an increase of the local temperature (within a range lying well below the glass transition temperature). This moderate temperature increase allows the thermal activation of the migration of silver ions as well as the chemical reactivity leading to the formation of a larger number of silver clusters^20^ which results in a higher ∆n. The same scenario applies for increasing the laser irradiance, the higher the irradiance the greater the formation of silver clusters as previously reported^14^. The ∆n increases from 0.9 × 10^−3^ up to 8 × 10^−3^ in this case (Fig. 3), which is to be compared to the ∆n values obtained in fused silica going from 1.3 × 10^−3^ up to 1.7 × 10^−2^ with *type I* modification^24, 25^.

To further support the hypothesis that this ∆n is related to the creation of silver clusters, a non-silver containing zinc phosphate glass was irradiated using identical irradiation conditions and even higher irradiance. Neither fluorescence nor refractive index change (∆n) was observed in this case, which indicates that the formation of silver clusters is the main and direct cause for refractive index change in silver containing zinc phosphate glass, in the low pulse energy regime. Indeed, we claim that our index change is not based on a standard index modification of the glass matrix itself, due to laser-induced modifications of the local density as it is the case for *type I*. Here, we report that the produced ∆n is based on the localized laser-induced creation of new chemical silver species with enlarged molecular electric polarizability, locally leading to an increase of the electric susceptibility and associated refraction index change. These considerations allow us to classify this silver-supported index change as a new type of refractive index change, being totally independent from index modification of the glass matrix itself. Indeed, the absence of laser-induced modifications in a same non-containing silver glass matrix ensures (i) that the corresponding ranges of irradiance and number of pulses do not affect the glass matrix itself and (ii) that the laser-matter energy deposition occurs through multi-photon ionization of the silver ions. Therefore, the formation of silver clusters occurs for pulse energies lower than the onset of the *type I* modification in this glass. The involved DLW process corresponds to an extrinsic laser structuring since it fully preserves the glass matrix while acting on the incorporated silver ions. In order to illustrate the potential of this new DLW process for practical applications, we have inscribed waveguiding structures in the bulk of our photosensitive glass. A series of 7 mm long straight waveguides were written 160 µm below the surface, while the laser parameters were varied. To get a single mode waveguide, the laser irradiance as well as the number of pulses were both optimized to get the appropriate size and ∆n.

**Figure 4:**
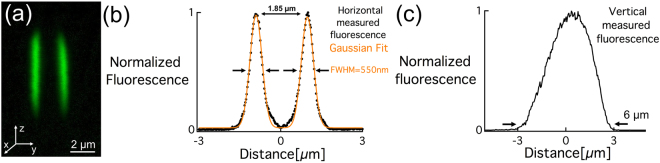
(**a**) Fluorescence confocal image of the side view of the waveguide, (**b**) normalized horizontal fluorescence profile averaged along the z axis and (**c**) vertical fluorescence normalized profile averaged along the y axis.

The structures/waveguides were imaged using a confocal fluorescence microscope (side view, Fig. 4a), which provides a better resolution of the silver cluster spatial distribution. For the sake of simplicity, one typical waveguide was fully characterized. The photo-induced structures consisted of two parallel fluorescent planes exhibiting a full width half maximum (FWHM) of 0.55 µm (limited by confocal microscope resolution which is typically 300 nm in this glass) and spaced by 1.85 µm along the y direction (Fig. 4.b). The corresponding length of the twin structure (along z) was estimated to be 6 μm (Fig. 4.c). However, we note that the spacing between the two parallel fluorescent planes as well as their extent along z could both be varied from 1.8 µm to 3 µm and 4 µm to 15 µm respectively, depending on the microscope objective and the laser parameters used for writing.

**Figure 5:**
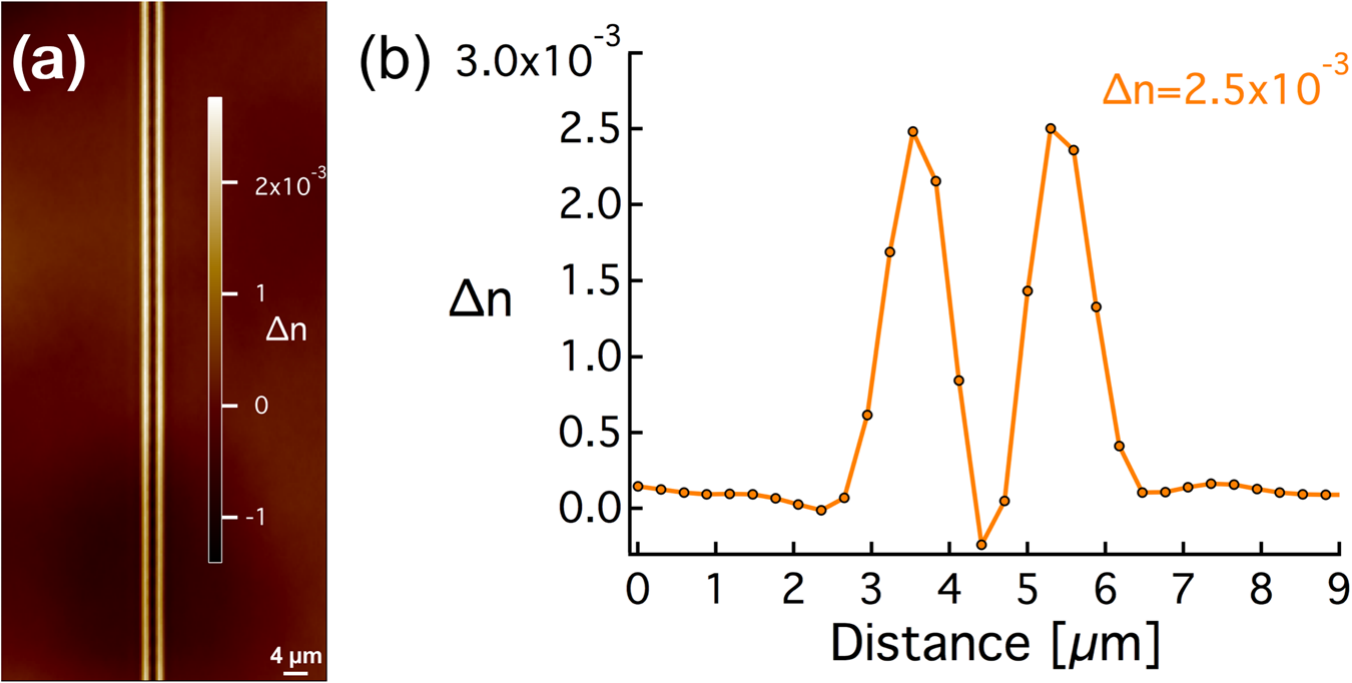
(**a**) Phase contrast image under while light illumination centered around 550 nm (top view) and (**b**) associated refractive index profile of a linear trace of Ag_m_^x+^ clusters. DLW conditions: 9 TW/cm^2^–60 µm/s.

The refractive index profile of the traces of photo-induced clusters was also measured using a quantitative phase camera from PHASICS, as explained in the methods section. It exhibits a smooth and homogeneous aspect as well as a sharp spatial distribution as shown in Fig. 5.a. The transverse profile shows two peaks of positive refractive index change ∆n of 2.5 × 10^−3^ spaced by ~1.8 µm and with a FWHM of approximately 0.8 μm (Fig. 5.b). Note that the measured FWHM of 0.8 μm is limited by the instrument spatial resolution. The good matching between the shape of the fluorescence confocal image (Fig. 4.b) and the Δn profile (Fig. 5.b) are confirming that the spatial distribution of the ∆n is indeed correlated to the spatial distribution of the silver clusters.

**Figure 6:**
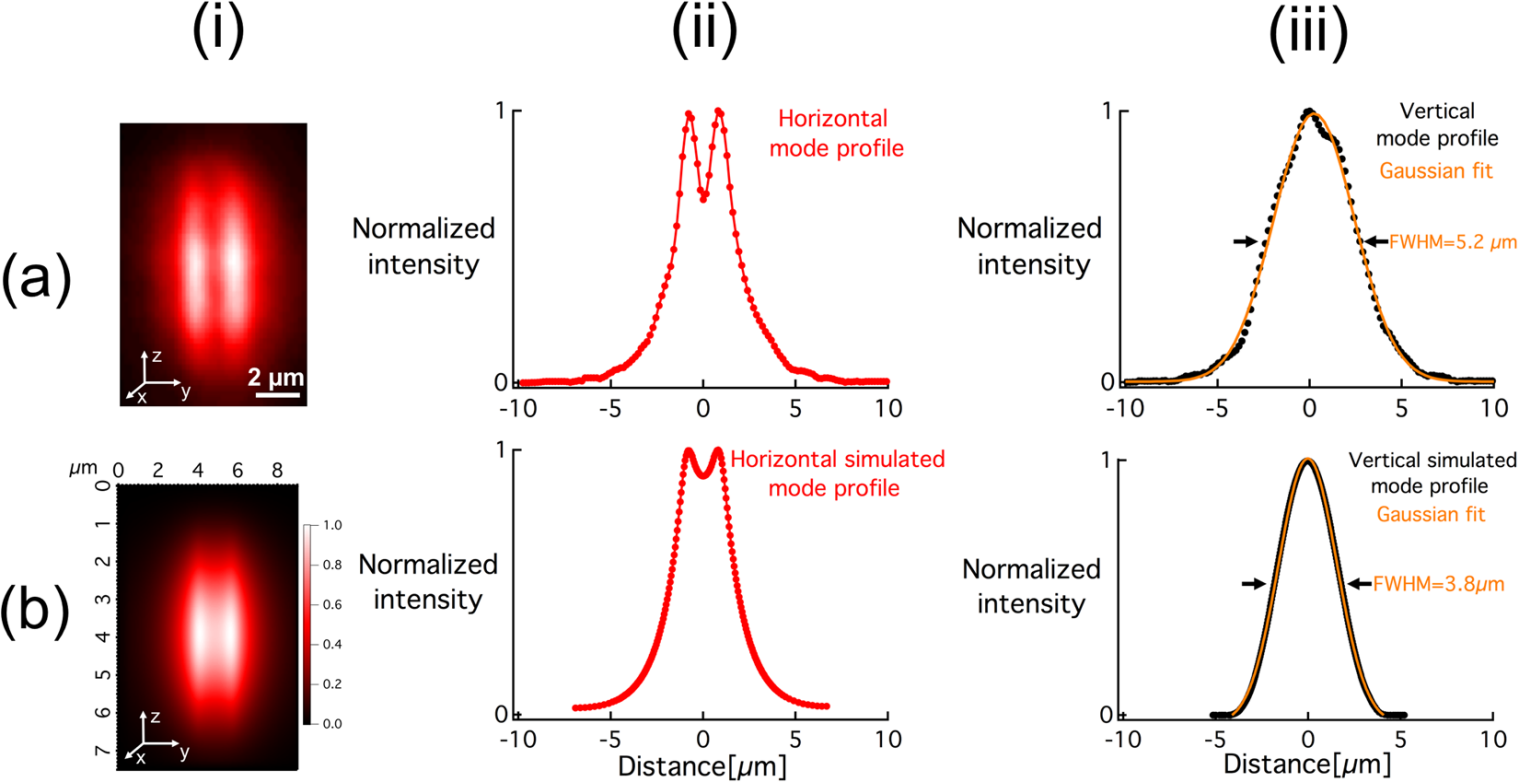
(**a**.i) Near-field mode profile after injecting a 630 nm laser, (**a**.ii) normalized horizontal mode profile averaged along the z axis, and (**a**.iii) normalized vertical mode profile averaged along the y axis. (**b**.i) Simulated mode profile (to compare with Fig. 6.a.i), (**b**.ii) normalized horizontal mode profile averaged along the z axis (to compare with Fig. 6.a.ii), and (**b**.iii) normalized vertical mode averaged along the y axis.

Preliminary waveguiding behavior of the photo-induced silver clusters Ag_m_^x+^ was first reported recently by Danto *el al* ^23^. using a CW NIR laser emitting at λ = 1030 nm, but without any detailed analysis of the waveguiding properties. In this report, we investigate this aspect by injecting a CW laser emitting at 630 nm inside the structures. The near-field intensity profile at the output of the waveguide (Fig. 6.a.i) was imaged using a CCD camera and a microscope objective (see Methods section hereafter).

The horizontal intensity mode profile depicted in Fig. 6.a.ii is a non-standard single-mode profile that can be fitted by the superposition of two Gaussian distributions (FWHM = 3 µm) while the vertical intensity mode profile shows a good match with a Gaussian fit as expected (FWHM = 5.2 µm) (Fig. 6.a.iii). Based on numerical simulations, each single laser-induced trace made of silver clusters Ag_m_^x+^ is expected to support waveguiding. In the case of the experimental structures with two Ag_m_^x+^ traces exhibiting a small separation distance (~1.85 µm) and a relatively low ∆n (~2.5 × 10^−3^), the horizontal intensity mode profile (Fig. 6.a.ii) was observed to remain the same, whatever the injection condition. Indeed, the spatial overlap of the injecting fiber with the guiding structure only influenced the transmitted intensity of the guided mode, without affecting its transverse output profile (which was not the case in multi-mode waveguides, not presently here for the sake of clarity). Therefore, this allows us to conclude that the considered guiding structure supports only a single mode (at λ = 630 nm), despite its non-standard profile. Such original double-trace structure, supported by the laser-induced silver clusters, seems thus to behave similarly to two close and highly interacting waveguides^26, 27^, globally corresponding to a structure that sustains a single super mode. Although as single-mode, one can still note that such waveguide is elliptical (${\rm{\varepsilon }}$ = 1.7).

Furthermore, simulations were conducted to correlate the experimental and simulated near-field mode profiles. The simulated mode in Fig. 6.b.i shows a significant match with the experimental mode, which supports our claim concerning the new type of refractive index change based on silver clusters formation. Furthermore, it confirms the correlation between the fluorescence i.e. silver clusters spatial distribution (Fig. 4.a) and the ∆n (Fig. 5). However, we noticed a difference between the dimensions of the experimental and simulated mode (Fig. 6). This is most likely due to the step index ∆n used for simulations (instead of a smooth distribution of index change in the structured glass) and the fact that the waveguide is simulated as two ellipsoids with positive ∆n, which slightly differs from the real gradient of the index change and from the real shape of the waveguide.

Finally, a large mismatch between the injected mode profile and the geometry of the waveguide results in injection losses of 5.22 dB, as estimated by the numerical simulations. That is also related to the fact that the waveguide is not circular but we can illustrate it as two ellipsoids with positive ∆n spaced by ~1.85 µm (see methods). The overall propagation losses of 1.2 dB/cm are the upper bound, estimated considering the simulated mismatch, Fresnel losses of 0.43 dB (n = 1.59) and the measured insertion losses of the 7-mm waveguide (see methods). Also, in our case using the traditional methods for characterizing propagation losses is not highly efficient due to the large mismatch between the fiber and the waveguide geometry, and the short length of the waveguide. Further efforts are required to get an accurate evaluation of the propagation losses of such waveguides.

**Figure 7:**
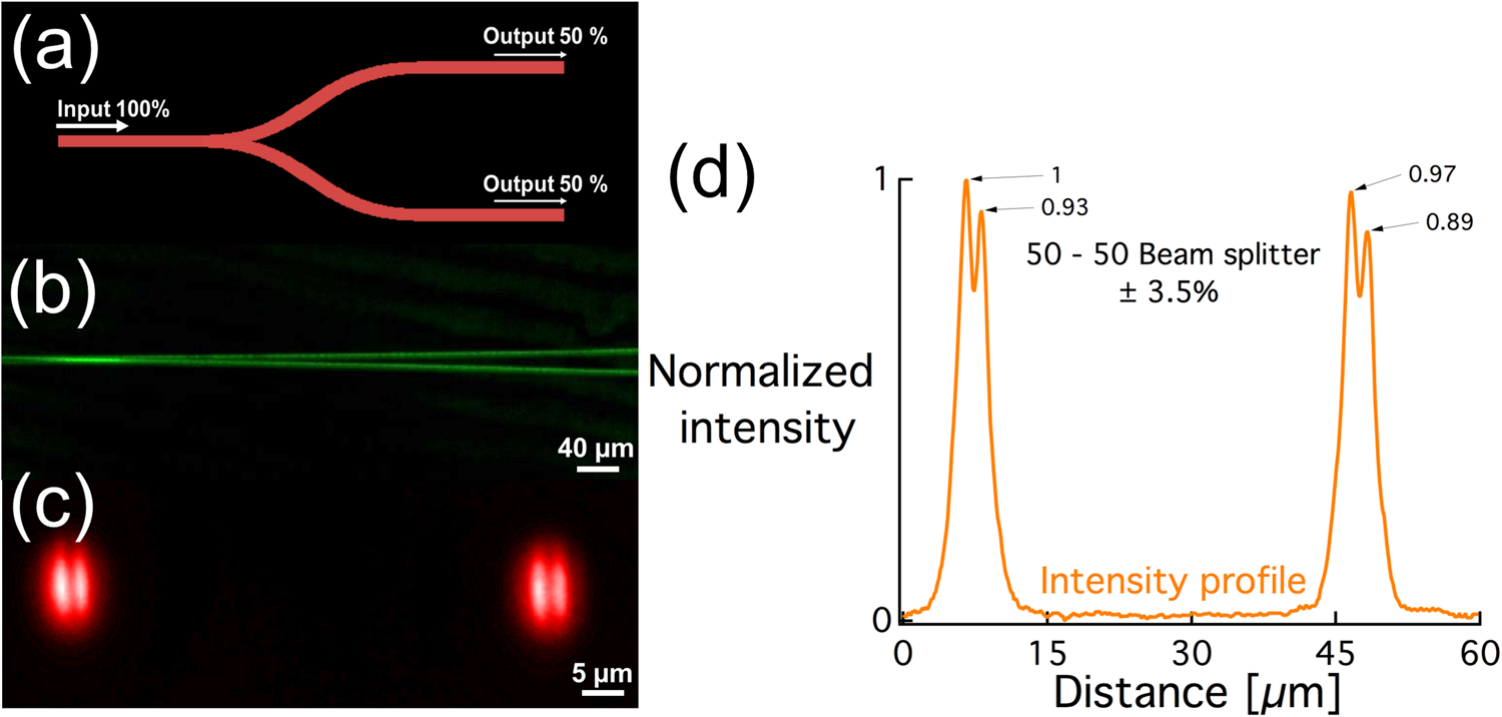
(**a**) Schematic presentation of a 50–50 beam splitter (top view), (**b**) fluorescent image (top view) of the written 50–50 beam splitter under 488 nm excitation and emission in the visible range, (**c**) output modes of the beam splitter, and (**d**) normalized intensity profile of the output modes. DLW conditions: 9 TW/cm^2^–60 µm/s.

Once the waveguiding properties were investigated and confirmed, we moved on to create an optical component to take a step forward towards applications such as 3D integrated circuits. A 50–50 y-shaped beam splitter (Fig. 7.a) was inscribed 160 µm below the surface of a silver-containing zinc phosphate glass sample (4% molar of Ag_2_O), by sequentially writing two partially overlapping S-bended waveguides. The optical component divides the input light power equally through each of the two output branches (Fig. 7.a). Fluorescence image of the written beam splitter is shown in Fig. 7.b. Following laser injection in the beam splitter, the output facet of the sample was imaged and two identical spots, as depicted in Fig. 7.c indicating that the input beam was split into two different output branches. The near-field intensity profile shows that the transmitted power is equal in both branches within an error margin of 3.5% (Fig. 7.d) confirming the inscription of a 50–50 beam splitter.

Finally, DLW was also conducted on silver containing zinc phosphate ribbon fibers. Our group previously reported the fabrication of photosensitive silver-containing zinc phosphate flat substrate fibers and the capabilities of performing DLW on such fibers^23^. Moreover, DLW of schemes like Mach-Zender interferometers and a ring resonator were performed in ribbon fibers but light was not injected in such devices yet. We claimed that the integrity of the glass was preserved from bulk samples to ribbon fibers. Hereby, we confirm our claim by writing a 1.4 cm waveguide inside a *500 µm × 200 µm* ribbon fiber, at 50 µm below the surface. After the inscription process, the ribbon fiber was cut and the end facets were polished to optical quality (see Methods). The fluorescent structures composed of silver clusters exhibit the same morphology as those of the structures written in bulk glasses, which also demonstrates the equivalence of the index modification profiles in bulk and fiber-shaped samples. A 630 nm laser was injected inside the 1.4 cm waveguide and the near field-mode profile was imaged.

Light is guided over the full length of the waveguide and the near-field intensity profile is similar to the profile of waveguides inscribed in bulk sample. The normalized horizontal and vertical intensity profiles in the ribbon fiber are much the same to the one observed in bulk glasses which are not shown in this paper for the sake of similarity.

Therefore, we demonstrate the capability of creating waveguides in ribbon fibers, thus paving the way for fiber applications in spectroscopy sensing, filtering and/or fully integrated photonics. DLW in silver-containing zinc phosphate bulk glasses and ribbon fibers allows the formation of a perennial new type of waveguides which take advantage of the thermal stability and bleaching-free fluorescence of the photo-induced silver clusters up to the glass transition temperature (typically 380–400 °C here)^28, 29^. Furthermore, such laser-induced modifications had already led to original second-order effective nonlinearities by means of direct laser poling, allowing for a localized second-harmonic generation that showed a significant efficiency with an effective coefficient close to the pm/V level^30^. In addition, a post laser irradiation thermal treatment (T_g_ + 20 °C) in silver-containing zinc phosphate glass had allowed the growth of silver metallic nanoparticles exhibiting an effective dielectric/metal medium bearing a plasmonic resonance response in the structured glass matrix^31^. To the best of our knowledge, combining optical and plasmonic waveguiding in a same glass sample is not achievable otherwise, which highlights the novelty and tremendous potential of these new type waveguides. Finally, one must emphasize that the creation of silver clusters is not only limited to the silver-containing zinc phosphate glasses but can also be generated in other silver containing glass matrixes such as sodocalcique and borosilicate glasses as previously reported by our group^22^. This proves the universality in terms of processes when considering tailored materials as silver-containing photosensitive glasses^32^, and the novelty of this technique in making hybrid integrated circuits in glasses.

## Conclusion

As a conclusion, we have demonstrated that direct laser writing (DLW) in silver-containing zinc phosphate glasses produces a novel type of positive refractive index change (∆n) that is compatible for waveguiding applications. This ∆n differs from the standard *type I* modification as it takes origin in the creation of the photo-induced silver clusters around the interaction voxel, involving such new chemical bonds with more polarizable electron clouds than those of the initial ions Ag^+^, and this leading to a local refractive index increase independently from any modification of the glass matrix itself. A 3 × 6 µm^2^ single-mode waveguide was written inside bulk glass, exhibiting a ∆n of 2.5 × 10^−3^, a single mode profile supported by each of the two silver clusters traces and showing an upper propagation losses of 1.2 dB/cm at λ = 630 nm. Moreover, a 50–50 beam splitter was written paving the way for 3D integrated optical circuits in such glasses. Finally, a 1.4 cm waveguide was inscribed inside ribbon fibers of these silver-containing zinc phosphate glass, demonstrating the waveguiding properties of such fibers once they are structured. These findings provide unique opportunities for applications such as spectroscopy fiber sensing, filtering and/or fully integrated photonics.

## Methods

### Glass Synthesis

A 0.4 P_2_O_5_ – 0.55ZnO – 0.01Ga_2_O_3_ – 0.04 Ag_2_O glass was elaborated from a mix of powders Zn(PO_3_)_2_, ZnO, Ga_2_O_3_, and Ag(NO_3_). Glass samples were made using a melt-quenching technique: a platinum crucible containing the powders was heated at 1200 °C during 12 h. The samples were quenched, ground and melted twice with the intention of improving the chemical homogeneity of the glass and then limit the local refraction index variations in order to get the best optical quality. Afterwards, the samples were annealed at 40 °C below the glass transition for 4 h to relax the accumulated mechanical constraints. Then samples were cut and polished for DLW. Moreover, after DLW, the facets were cut and polished again to reveal the waveguides to the surface, to allow laser injection and the visualization of the guided mode profile. Preforms for fiber drawing were produced by casting the glass in a specifically designed brass mold, pre-heated at ~T_g_-10 °C and annealed at T_g_-40 °C for 12 hours.

### Ribbon Fiber Drawing

Thermal drawing was performed using a dedicated 3-meter-high optical fiber draw tower composed of an annular electrical furnace with a sharp temperature profile, a diameter monitor, a tension dancer and a collecting drum. The rectangular preform was slowly fed into the furnace and the temperature was gradually increased up to ~700 °C under continuous oxygen gas flow (3 l.min^−1^). The preform-holder motion and capstan rotation velocity were controlled in real-time to produce the targeted fiber diameter. Following this procedure, meters of fibers were drawn with dimensions ranging from 600 µm × 300 µm, down to 200 µm × 100 µm.

### 3D Direct Laser Writing

The glass samples were irradiated using a Yb:KGW femtosecond oscillator (Amplitude system, up to 2.6 W average power, 9.8 MHz repetition rate, 390 fs pulse duration (FWHM) and emitting at 1030 nm). The laser irradiance was controlled with an acousto-optic modulator enabling the accumulation of N = 10^5^–10^6^ pulses with energy pulses going from 20 nJ up to 150 nJ. The sample displacement and positioning was carried out using a high precision 3D translation stage (XMS-50 stages, Micro controller). Direct laser writing was performed using a microscope objective (Carl Zeiss, 20x - 0.75NA) and all the structures were written 160 µm below the surface except for the ribbon fibers.

### Bulk Glasses

The cartographies of square linear structures were written using irradiances going from 6 TW/cm^2^ to 10 TW/cm^2^ while the writing speed varied between 10 µm/s and 50 µm/s corresponding to 1.7 × 10^6^ deposited pulses and 3.3 × 10^5^ deposited pulses respectively.

The waveguide and the beam splitter were written using 9 TW/cm^2^ laser irradiance and 60 µm/s writing speed. The beam splitter consisted of two partially-overlapping opposite s-bend waveguides.

### Ribbon fibers

The waveguide was written 50 µm below the surface using an irradiance of 10 TW/cm^2^ and a writing speed of 25 µm/s. Following the waveguide inscription, the fiber facets were carefully polished to reveal the waveguide to the surface allowing laser injection and the mode profile imaging.

### Mode Profile Setup

Near field mode profiles were observed after injecting light of a red diode laser emitting at 630 nm using a single mode HP-460 fiber (core diameter 2.5 µm - NA = 0.13) into the waveguides and beam splitter. Then, a 100x – 0.55 NA Mitutoyo objective was focused on the output facet to visualize the guided mode profile and conjugate it on a CCD camera.

### Loss estimation

The losses were estimated by measuring the power of injected light in the waveguide and the power transmitted. Moreover, the mode mismatch between the fiber and the waveguide was taken into consideration as well as Fresnel losses. The mode mismatch was simulated using Lumerical software. A power coupling coefficient between the HP-460 fiber and the waveguide was simulated to be ~30% i.e. 5.22 dB of loss in ideal conditions. Fresnel losses were calculated to 0.43 dB corresponding to the two glass facets. Finally, taking into consideration the simulated mismatch, Fresnel losses and the measured insertion losses, the higher bond of the propagation losses was estimated to be 1.2 dB/cm.

### Numerical simulations

Simulations of the guided mode were conducted using Lumerical’s Mode solutions software. The waveguide geometry was simulated based on the confocal fluorescence images and the refractive index change measurement. The simulations consisted of two parallel ellipsoids of 550 nm thickness, spaced by 1.85 µm, with a depth of 6 µm (z axis) and a step index refractive index change of 2.5 × 10^−3^. The propagation of a 630 nm beam was simulated inside the above-mentioned structure.

### Fluorescence confocal images

Were obtained using an Olympus FLUOVIEW FV1200 confocal microscope. An oil immersion objective of 60x was used to resolve the structures. Excitation wavelength was at 480 nm while the emission wavelength was broad band between 500 nm and 600 nm.

### ∆n measurement

Refractive index variation (∆n) between the unmodified glass and the silver clusters was measured using a phase-contrast microscopy method with commercially SID4Bio available equipment SID4Bio from PHASICS Inc. A 100x – 1.3 NA oil immersion objective was used to image the structures using white light, typically leading to 800 nm transverse spatial resolution (FWHM).

### Data Availability

The datasets generated during and/or analyzed during the current study are available from the corresponding author on reasonable request.
